# Supplementary material for: Improving the Sexual Health of Young People (under 25) in High-Risk Populations: A Systematic Review of Behavioural and Psychosocial Interventions
Source: Int J Environ Res Public Health. 2021 Aug 27;18(17):9063. doi: 10.3390/ijerph18179063 (PMC8430747; doi:10.3390/ijerph18179063)
Supplement: Supplementary file 1 [file ijerph-18-09063-s001.zip › ijerph-1298552-supplementary.pdf]

MEDLINE (Ovid) Search Terms

1 (("safe sex" or "unsafe sex" or "sexual risk taking" or "sex* risk behavio*r" or STI or HIV or chlamydia or gonorrhoea or hepatitis or syphilis or "acquired immunodeficiency syndrome" or condom* or "barrier contraception" or "unwanted pregnancy").ti,ab. or exp *Sexually Transmitted Diseases/ or exp *Sexual Health/ or exp *Sexual Behavior/ or exp *Sex Education/ or exp *Sex Education/mt or exp *Health Knowledge, Attitudes, Practice/ or exp *Contraception, Barrier/ or exp *Contraception Behavior/ or exp *Contraception/ or exp *Long-Acting Reversible Contraception/ or exp *Hormonal Contraception/ or exp *Pregnancy, Unwanted/) and (("behavio* therapy" or "cognitive therapy" or "psychosocial intervention" or "skills training" or "sex education" or "motivational interview*").ti,ab. or exp *Psychosocial Intervention/ or exp *Behavior Therapy/ or exp *Cognitive Behavioral Therapy/ or exp *Motivational Interviewing/) and ((youth or "young adult*" or teenage* or "high school student*" or adolescen* or "university student*".ti,ab.) or exp *Adolescent/ or exp *Young Adult/ or exp *Child/ or exp *Students/ or exp *Adolescent Behavior/)

2 (alcohol* or binge or ethanol or drink* or "drug use" or "recreational drug*" or "substance use").ti,ab. or exp *Alcohol Drinking/ or exp *Substance-Related Disorders/ or exp *Alcoholism/ or exp *Illicit Drugs/

3 ("minority group*" OR "ethnic minorit*" OR "ethnic group*" OR immigrant* OR "racial group*" OR black OR hispanic OR latin* OR asian OR indigenous OR "native American" OR aboriginal OR "first nations" OR african OR vietnamese OR "hawaii* native" OR asian OR indian OR inuit OR "pacific islander*" OR "american indian*").ti,ab. or exp *Ethnic Groups/ or exp *Minority Groups/ or exp *Indigenous Canadians/ or exp *Health Services, Indigenous/ or exp *Indigenous Peoples/

4 (homeless* or "street youth" or "runaway youth").ti,ab. or exp *Homeless Youth/

5 ("juvenile justice" or probation or court* or "law enforcement" or "diversion program" or "juvenile detention").ti,ab. or exp *Juvenile Delinquency/

6 (gay or lesbian* or genderqueer or transgender* or homosexual* or bisexual or bicurious or "female to male" or "male to female" or trans or "men who have sex with men" or MSM or intersex or LGBT or LGBTQ or LGBTQI or "gender transition" or "gender dysphoria" or "gender identity" or "same sex attracted" or "same sex couple" or "same sex couples" or "sexual and gender minorities" or "sexual orientation" or "sexual preference" or "trans wom#n" or "trans m#n" or "trans people" or "women loving women" or "women who have sex with women" or WSW).ti,ab. or exp *"Sexual and Gender Minorities"/

7 ("severe mental illness" or "serious mental illness" or "chronic mental illness" or schizophrenia or bipolar or mania or psychosis or schizoaffective or "major depressive disorder" or MDD).ti,ab. or exp *Mental Disorders/

8 ("foster care" or "group home*" or "out-of-home care" or "residential care").ti,ab. or exp *Foster Home Care/

9 2 or 3 or 4 or 5 or 6 or 7 or 8

10 1 and 9

PSYCInfo (Ovid) Search Terms

1 (("safe sex" or "unsafe sex" or "sexual risk taking" or "sex* risk behavio*r" or STI or HIV or chlamydia or gonorrhoea or hepatitis or syphilis or "acquired immunodeficiency syndrome" or condom* or "barrier contraception" or "unwanted pregnancy").ti,ab. or exp *Sexually Transmitted Diseases/ or exp *Sexual Health/ or exp *Sexual Behavior/ or exp *Sex Education/ or exp *Contraception, Barrier/ or exp *Contraception Behavior/ or exp *Contraception/ or exp *Long-Acting Reversible Contraception/ or exp *Hormonal Contraception/ or exp *Pregnancy, Unwanted/) and (("behavio* therapy" or "cognitive therapy" or "psychosocial intervention" or "skills training" or "sex education" or "motivational interview*").ti,ab. or exp *Psychosocial Intervention/ or exp *Behavior Therapy/ or exp *Cognitive Behavioral Therapy/ or exp *Motivational Interviewing/) and ((youth or "young adult*" or teenage* or "high school student*" or adolescen*).mp. or "university student*".ti,ab. or exp *Adolescent/

or exp *Young Adult/ or exp *Child/ or exp *Students/ or exp *Adolescent Behavior/) [mp=title, abstract, heading word, table of contents, key concepts, original title, tests & measures, mesh]

2 (alcohol* or binge or ethanol or drink* or "drug use" or "recreational drug*" or "substance use").ti,ab. or exp *Substance-Related Disorders/ or exp *Alcoholism/ or exp *Illicit Drugs/

3 ("minority group*" or "ethnic minorit*" or "ethnic group*" or immigrant* or "racial group*" or black or hispanic or latin* or asian or indigenous or "native American" or aboriginal or "first nations" or african or vietnamese or "hawaii* native" or asian or indian or inuit or "pacific islander*" or "american indian*").ti,ab. or exp *Ethnic Groups/ or exp *Minority Groups/ or exp *Health Services, Indigenous/ or exp *Indigenous Peoples/

4 (homeless* or "street youth" or "runaway youth").ti,ab.

5 ("juvenile justice" or probation or court* or "law enforcement" or "diversion program" or "juvenile detention").ti,ab. or exp *Juvenile Delinquency/

6 (gay or lesbian* or genderqueer or transgender* or homosexual* or bisexual or bicurious or "female to male" or "male to female" or trans or "men who have sex with men" or MSM or intersex or LGBT or LGBTQ or LGBTQI or "gender transition" or "gender dysphoria" or "gender identity" or "same sex attracted" or "same sex couple" or "same sex couples" or "sexual and gender minorities" or "sexual orientation" or "sexual preference" or "trans wom#n" or "trans m#n" or "trans people" or "women loving women" or "women who have sex with women" or WSW).ti,ab.

7 ("severe mental illness" or "serious mental illness" or "chronic mental illness" or schizophrenia or bipolar or mania or psychosis or schizoaffective or "major depressive disorder" or MDD).ti,ab. or exp *Mental Disorders/

8 ("foster care" or "group home*" or "out of home care" or "residential care").ti,ab.

9 2 or 3 or 4 or 5 or 6 or 7 or 8

10 1 and 9

EMBase (Ovid) Search Terms

1 (("safe sex" or "unsafe sex" or "sexual risk taking" or "sex* risk behavio*r" or STI or HIV or chlamydia or gonorrhoea or hepatitis or syphilis or "acquired immunodeficiency syndrome" or condom* or "barrier contraception" or "unwanted pregnancy").ti,ab. or exp *Sexually Transmitted Diseases/ or exp *Sexual Health/ or exp *Sexual Behavior/ or exp *Sex Education/ or exp *Health Knowledge, Attitudes, Practice/ or exp *Contraception, Barrier/ or exp *Contraception Behavior/ or exp *Contraception/ or exp *Long-Acting Reversible Contraception/ or exp *Hormonal Contraception/ or exp *Pregnancy, Unwanted/) and (("behavio* therapy" or "cognitive therapy" or "psychosocial intervention" or "skills training" or "sex education" or "motivational interview*").ti,ab. or exp *Behavior Therapy/ or exp *Cognitive Behavioral Therapy/ or exp *Motivational Interviewing/) and ((youth or "young adult*" or teenage* or "high school student*" or adolescen*).mp. or "university student*".ti,ab. or exp *Adolescent/ or exp *Young Adult/ or exp *Child/ or exp *Students/ or exp *Adolescent Behavior/) [mp=title, abstract, heading word, drug trade name, original title, device manufacturer, drug manufacturer, device trade name, keyword, floating subheading word, candidate term word]

2 (alcohol* or binge or ethanol or drink* or "drug use" or "recreational drug*" or "substance use").ti,ab. or exp *Alcohol Drinking/ or exp *Substance-Related Disorders/ or exp *Alcoholism/ or exp *Illicit Drugs/

3 ("minority group*" or "ethnic minorit*" or "ethnic group*" or immigrant* or "racial group*" or black or hispanic or latin* or asian or indigenous or "native American" or aboriginal or "first nations" or african or vietnamese or "hawaii* native" or asian or indian or inuit or "pacific islander*" or "american indian*").ti,ab. or exp *Ethnic Groups/ or exp *Minority Groups/ or exp *Health Services, Indigenous/ or exp *Indigenous Peoples/

4 (homeless* or "street youth" or "runaway youth").ti,ab. or exp *Homeless Youth/

5 ("juvenile justice" or probation or court* or "law enforcement" or "diversion program" or "juvenile detention").ti,ab. or exp *Juvenile Delinquency/

6 (gay or lesbian* or genderqueer or transgender* or homosexual* or bisexual or bicurious or "female to male" or "male to female" or trans or "men who have sex with men" or MSM or intersex or LGBT or LGBTQ or LGBTQI or "gender transition" or "gender dysphoria" or "gender identity" or "same

sex attracted" or "same sex couple" or "same sex couples" or "sexual and gender minorities" or "sexual orientation" or "sexual preference" or "trans wom#n" or "trans m#n" or "trans people" or "women loving women" or "women who have sex with women" or WSW).ti,ab. or exp *"Sexual and Gender Minorities"/

7 ("severe mental illness" or "serious mental illness" or "chronic mental illness" or schizophrenia or bipolar or mania or psychosis or schizoaffective or "major depressive disorder" or MDD).ti,ab. or exp *Mental Disorders/

8 ("foster care" or "group home*" or "out of home care" or "residential care").ti,ab. or exp *Foster Home Care/

9 2 or 3 or 4 or 5 or 6 or 7 or 8

10 1 and 9

Web of Science Search Terms

(TS=("safe sex" OR "unsafe sex" OR "sexual risk taking" OR "sex* risk behavio*r" OR STI OR HIV OR chlamydia OR gonorrhoea OR hepatitis OR syphilis OR "acquired immunodeficiency syndrome" OR condom* OR "barrier contraception" OR "unwanted pregnancy") AND TS=("behavio* therapy" OR "cognitive therapy" OR "psychosocial intervention" OR "skills training" OR "sex education" OR "motivational interview*") AND TS=(youth OR "young adult*" OR teenage* OR "high school student*" OR adolescen* OR "university student*") AND ((TS=("severe mental illness" OR "serious mental illness" OR "chronic mental illness" OR schizophrenia OR bipolar OR mania OR psychosis OR schizoaffective OR "major depressive disorder" OR MDD)) OR (TS=(alcohol* OR binge OR ethanol OR drink* OR "drug use" OR "recreational drug*" OR "substance use")) OR (TS=("juvenile justice" OR probation OR court* OR "law enforcement" OR "diversion program" OR "juvenile detention")) OR (TS=(homeless* OR "street youth" OR "runaway youth")) OR (TS=(gay OR lesbian* OR genderqueer OR transgender* OR homosexual* OR bisexual OR biocurious OR "female-to-male" OR "male-to-female" OR trans OR "men who have sex with men" OR MSM OR intersex OR LGBT OR LGBTQ OR LGBTQI OR "gender transition" OR "gender dysphoria" OR "gender identity" OR "same sex attracted" OR "same sex couples" OR "sexual and gender minorities" OR "sexual orientation" OR "sexual preference" OR "trans wom*n" OR "trans m*n" OR "trans people" OR "women loving women" OR "women who have sex with women" OR WSW)) OR (TS=("foster care" OR "group home*" OR "residential care" OR "out of home care")) OR (TS=("minority group*" OR "ethnic minorit*" OR immigrant* OR "racial group*" OR hispanic OR latin* OR asian OR indigenous OR native american OR aboriginal OR "first nations" OR african OR vietnamese OR hawaii* native OR asian OR indian OR inuit OR "pacific islander*" OR "american indian*"))))

Scopus Search Terms

(TITLE-ABS-KEY(("safe sex" OR "sexual health" OR "sexual health promotion" or "sexual wellbeing" OR "unsafe sex" OR "sexual risk taking" OR "sex* risk behavio*" OR STI OR HIV OR chlamydia OR gonorrhoea OR hepatitis OR syphilis OR "acquired immunodeficiency syndrome" OR condom* OR "barrier contraception" OR "unwanted pregnancy" OR "pregnancy prevention") AND ("behavio* therapy" OR "cognitive therapy" OR "psychosocial intervention" OR "skills training" OR "sex education" OR "motivational interview*") AND (youth OR "young adult*" OR teenage* OR "high school student*" OR adolescen* OR "university student*"))) AND (TITLE-ABS-KEY(("severe mental illness" OR "serious mental illness" OR "chronic mental illness" OR schizophrenia OR bipolar OR mania OR psychosis OR schizoaffective OR "major depressive disorder" OR MDD) OR (alcohol* OR binge OR ethanol OR drink* OR "drug use" OR "recreational drug*" OR "substance use") OR ("juvenile justice" OR probation OR court* OR "law enforcement" OR "diversion program" OR "juvenile detention") OR (homeless* OR "street youth" OR "runaway youth") OR (gay OR lesbian* OR genderqueer OR transgender* OR homosexual* OR bisexual OR bicurious OR "female to male" OR "male to female" OR trans OR "men who have sex with men" OR MSM OR intersex OR LGBT OR LGBTQ OR LGBTQI OR "gender transition" OR "gender dysphoria" OR "gender identity" OR "same sex attracted" OR "same sex couples" OR "sexual and gender minorities" OR "sexual orientation" OR "sexual preference" OR "trans wom?n" OR "trans m?n" OR "trans people" OR "women loving women" OR "women who have sex with women"

OR WSW) OR ("minority group*" or "ethnic minorit*" or "ethnic group*" or immigrant* or "racial group*" or black or hispanic or latin* or asian or indigenous or native american or aboriginal or "first nations" or african or vietnamese or hawaii* native or asian or indian or inuit or "pacific islander*" or "american indian*")) OR ("foster care" or "group home*" or "residential care" or "out of home care"))

Cochrane Library Search Terms

1 "safe sex" OR “sexual wellbeing” OR “sexual health” OR "unsafe sex" OR "sexual risk" OR STI OR HIV OR chlamydia OR gonorrhoea OR hepatitis OR syphilis OR condom* OR "barrier contraception" OR “pregnancy prevention”

2 ("behavior* therapy" OR "cognitive therapy" OR "psychosocial intervention" OR "skills training" OR "sex education" OR motivational NEXT interview*) AND randomized

3 youth OR young NEXT adult* OR teenage* OR "high school students" OR adolescen*

4 alcohol* OR binge OR ethanol OR drink* OR "drug use" OR recreational NEXT drug* OR "substance use"

5 "juvenile justice" OR probation OR court* OR "law enforcement" OR "diversion program" OR "juvenile detention"

6 homeless OR “street youth” OR “runaway youth”

7 gay OR lesbian* OR genderqueer OR transgender* OR homosexual* OR bisexual OR bicurious OR "female-to-male" OR "male-to-female" OR trans OR "men who have sex with men" OR YMSM OR intersex OR LGBT* OR "gender transition" OR "gender dysphoria" OR "gender identity" OR "same sex attracted" OR "same sex couples" OR "sexual and gender minorities" OR "sexual orientation" OR "sexual preference" OR trans NEXT wom*n OR trans NEXT m*n OR "trans people" OR "women loving women" OR "women who have sex with women" OR WSW

8 "minority group*" OR "ethnic minorit*" OR immigrant* OR "racial group*" OR hispanic OR latin* OR asian OR indigenous OR native american OR aboriginal OR "first nations" OR african OR vietnamese OR hawaii* native OR asian OR indian OR inuit OR "pacific islander*" OR "american indian*"

9 “foster care” OR “group home*” OR “residential care” OR “out of home care”

10 "severe mental illness" or "serious mental illness" or "chronic mental illness" or schizophrenia or bipolar or mania or psychosis or schizoaffective or "major depressive disorder" or MDD:ti,ab

1 AND 2 AND 3 AND (4 OR 5 OR 6 OR 7 OR 8 OR 9)
